# TransRAUNet: A Deep Neural Network with Reverse Attention Module Using HU Windowing Augmentation for Robust Liver Vessel Segmentation in Full Resolution of CT Images

**DOI:** 10.3390/diagnostics15020118

**Published:** 2025-01-07

**Authors:** Kyoung Yoon Lim, Jae Eun Ko, Yoo Na Hwang, Sang Goo Lee, Sung Min Kim

**Affiliations:** 1 Department of Medical Device and Healthcare, Dongguk University, Seoul 04620, Republic of Korea; thrtjr123@naver.com (K.Y.L.); yoona7747@gmail.com (Y.N.H.); gu2zzan@naver.com (S.G.L.); 2 Department of Regulatory Science for Medical Device, Dongguk University, Seoul 04620, Republic of Korea; 2018111721@dgu.ac.kr

**Keywords:** deep learning, liver vessel segmentation, CT dataset, convolution neural network, transformer, reverse attention module, Hounsfield unit windowing augmentation

## Abstract

**Background:** Liver cancer has a high mortality rate worldwide, and clinicians segment liver vessels in CT images before surgical procedures. However, liver vessels have a complex structure, and the segmentation process is conducted manually, so it is time-consuming and labor-intensive. Consequently, it would be extremely useful to develop a deep learning-based automatic liver vessel segmentation method. **Method:** As a segmentation method, UNet is widely used as a baseline, and a multi-scale block or attention module has been introduced to extract context information. In recent machine learning efforts, not only has the global context extraction been improved by introducing Transformer, but a method to reinforce the edge area has been proposed. However, the data preprocessing step still commonly uses general augmentation methods, such as flip, rotation, and mirroring, so it does not perform robustly on images of varying brightness or contrast levels. We propose a method of applying image augmentation with different HU windowing values. In addition, to minimize the false negative area, we propose TransRAUNet, which introduces a reverse attention module (RAM) that can focus edge information to the baseline TransUNet. The proposed architecture solves context loss for small vessels by applying edge module (RAM) in the upsampling phase. It can also generate semantic feature maps that allows it to learn edge, global context, and detail location by combining high-level edge and low-level context features. **Results:** In the 3Dricadb dataset, the proposed model achieved a DSC of 0.948 and a sensitivity of 0.944 in liver vessel segmentation. This study demonstrated that the proposed augmentation method is effective and robust by comparisons with the model without augmentation and with the general augmentation method. Additionally, an ablation study showed that RAM has improved segmentation performance compared to TransUNet. Compared to prevailing state-of-the-art methods, the proposed model showed the best performance for liver vessel segmentation. **Conclusions:** TransRAUnet is expected to serve as a navigation aid for liver resection surgery through accurate liver vessel and tumor segmentation.

## 1. Introduction

Liver resection is a surgical operation within the abdomen that inevitably leads to bleeding, and surgical difficulty can increase depending on the location of both liver vessels and tumors [1]. In particular, iatrogenic vascular damage due to limited visualization of major organs and blood vessels during surgical procedures is one of the most dangerous complications of surgical resection [2]. Therefore, it is important to identify the location of vessels destined to either be resected or preserved before surgery.

Computed Tomography (CT) is a common medical imaging modality used to visualize the vascular structure of a patient’s liver. CT imaging makes it easy to observe the location and size of organs and has the advantage of enhancing the anatomical position of vessels using contrast agents. Clinical experts acquire three-dimensional abdominal CT images and manually segment the liver vessels for each image slice. This task is a computational load and can be extremely labor-intensive depending on the environment, which includes variables such as noise, low contrast, complex structure of the liver, or radiologist skill [3]. Small hepatic vessels have relatively low imaging contrast, so there can be differences in segmentation results depending on clinical proficiency [4].

Traditional blood vessel segmentation methods that employ edge-based region growing have been proposed in the last few years. The region-based segmentation method is an algorithm that utilizes the similarity of features within the target region, which is simple to calculate but has the disadvantage of being sensitive to noise and CT intensity range [5,6,7,8,9,10]. The edge-based segmentation method that detects boundaries in different areas and enhances blood vessels using filters has the disadvantage of requiring complex parameter adjustment [11,12]. Meanwhile, the tracking method is an algorithm that tracks blood vessels based on the minimum cost path by tracking blood vessel trajectories, and if the initial seed point is incorrect, the segmentation results may appear to be unexpected [13,14,15].

Subsequently, CNN-based deep learning models such as FCN [16] and U-Net [17] have been applied for liver vascular segmentation within CT images. CNN-based segmentation models typically capture low-level spatial features but lack the ability to capture global context features. To overcome these limitations, Dense [18,19,20] Multiscale network [21], Attention gate [4], and Transformer based liver vessel segmentation models [22] were developed. Transformer converts images into one-dimensional sequences and focuses on global contexts through a Self-Attention mechanism, while generating low-resolution feature maps that lack detailed localization information. Although Transformer shows high performance in image classification tasks by capturing global context compared to CNN-based models, it is inadequate for obtaining detailed location information in segmentation tasks. Consequently, Chen et al. proposed TransUnet [23], which combines the features of CNN and Transformer models, and has shown high performance in multi-organ segmentation within abdominal CT images. However, it is still difficult to segment the detailed structure of liver vessels due to structurally complex backgrounds such as liver tumors or morphological variability in surrounding tissues [22]. Additionally, it is difficult to extract edge features during the upsampling process due to the small size of liver vessels.

Previous studies have identified two major limitations. First, although models have shown excellent segmentation performance for large blood vessels such as the aorta, they do not capture the edge features of small blood vessels very well. Second, the existing method trained the model through general augmentation methods such as rotating, flip, and mirroring. This is known to be effective in preventing overfitting and improving performance when using a limited amount of learning data. However, in actual clinical conditions rather than training conditions, robustness may be reduced for images of different brightness and contrast due to the varying conditions of imaging equipment and patient characteristics. Therefore, in this study, we have proposed TransRAUnet, which improves segmentation performance for the detailed structures of liver blood vessels and maintains robustness under various image quality conditions.

The main contributions of the paper are as follows:

1. Integration of a TransUnet-based global context extraction module, enabling precise segmentation of intricate liver blood vessel structures.
2. Implementation of a reverse attention module tailored to accurately capture and segment subtle edge features in small blood vessels.
3. Introduction of Multi-HU Windowing augmentation, a novel approach that synthesizes diverse image brightness conditions, enhancing the robustness and reliability of the model across varying clinical scenarios.

When trained through the proposed technique, the model can greatly alleviate overfitting to specific image brightness and contrasts and improve liver vessel segmentation performance by introducing edge features.

The paper is organized as follows: Section 2 shows related works on model construction and liver vessel segmentation in CT. Section 3 explains the details of the proposed method. Section 4 evaluates the performance results that were quantitatively and qualitatively obtained by implementing the proposed method. Section 5 presents a discussion, and, finally, Section 6 presents the conclusion.

## 2. Related Work

### 2.1. Model Construction

Ronneberger et al. proposed a U-shaped fully convolutional network called Unet [17]. Unet’s encoder is a contracting path that extracts context, and the decoder is an expanding path that extracts location information. Due to the characteristics of this structure, it is widely used as a baseline in medical image segmentation work, and elements such as multi-scale and attention modules have been introduced to improve performance [24,25]. For vessel segmentation in CT, patch-based 2D segmentation [26] and 3D segmentation using 3D-Unet are commonly used [27,28].

Vaswani et al. proposed a sequence-to-sequence architecture for machine translation in NLP tasks, called Transformer [29]. Much research has been conducted in efforts to apply Transformer’s powerful self-attention mechanism to medical image classification or segmentation tasks. Vision Transformer, for instance, showed superior performance in ImageNet classification compared to state-of-the-art networks [30]. TransUnet, proposed by Chen [23], is a U-Net hybrid of Transformer and CNN which not only has global context extraction and but also detail localization.

Huang et al. proposed a reverse attention method which has the characteristic of identifying a mixture of object and background areas and learning the differences [31]. Its principle is to identify the mixing between areas by inversely calculating the object area to the background area and has mainly been applied to object detection. Chen et al. detected salient objects by introducing reverse attention, which can help with top-down residual learning [32]. Li et al. achieved a Surface DSC of 0.739 in the COVID-19 segmentation task by applying Reverse Attention [33].

### 2.2. Deep Learning of Liver Vessel Segmentation in CT Images

In other approaches, Suvarachakan et al. achieved a DSC of 0.830 using an ensemble of models trained with various filter enhancement methods on CT images [34]. Yan et al. achieved a DSC of 0.805 by capturing multi-scale attention information from CT images and integrating low-level features with high-level features [4]. Meng et al. achieved an improved DSC of 0.921 in the liver vessel segmentation task with a model that combined Transformer, CNN, and Edge extraction module using Canny [22]. Wu et al. [35] introduced a U-shaped swin-Transformer-based multi-head attention vessel network, achieving a DSC of 0.775 in liver vessel segmentation. These studies have shown good performance; however, there are still limitations in effectively capturing the boundaries and small structures of vessels. Furthermore, the studies of liver vessel segmentation have been conducted only in certain brightness environments. This may hinder its generalization ability. Therefore, to deal with real world data, there is a pressing need to achieve accurate segmentation of detailed hepatic vascular structures under various brightness conditions and corresponding environments.

## 3. Method

**Figure 1:**
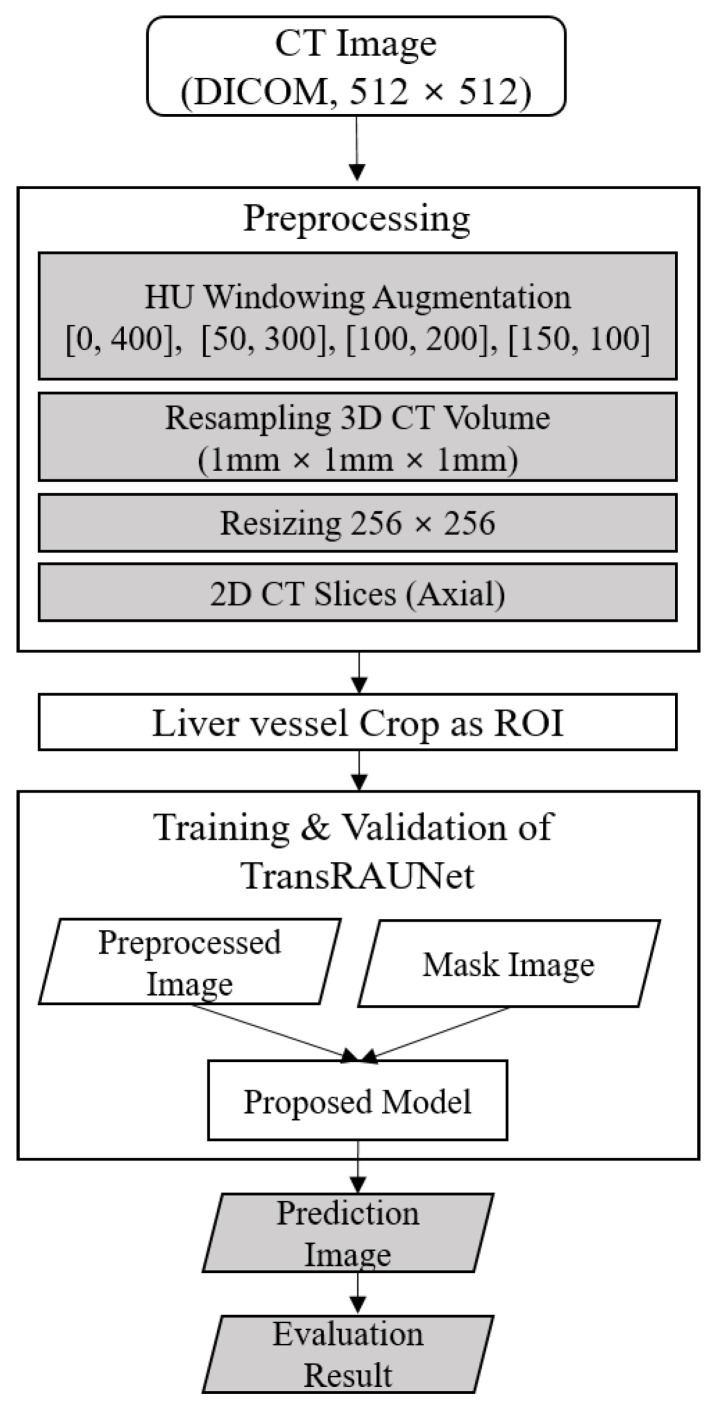
Flowchart of the proposed algorithm.

We proposed TransRAUNet with a multi-HU windowing augmentation method. The proposed model constructed TransUnet as a baseline and introduced a Reverse Attention Module (RAM) to focus on the edge features of liver vessels. In the encoder, a hybrid of CNN and Transformer, global context features are extracted through self-attention, and detailed hepatic vessel locations and edge features are extracted from the decoder with RAM. In addition, rather than the image augmentation approach commonly used in computer vision, we proposed an approach that enables robust learning for various image brightness by applying different HU windowing ranges applicable to the medical environment. Figure 1 shows the flowchart of the proposed algorithm.

### 3.1. HU Windowing-Based Image Augmentation

**Figure 2:**
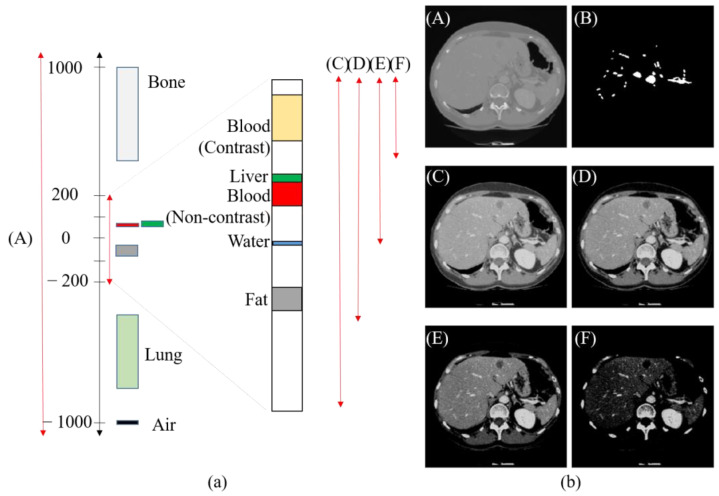
Data augmentation with various HU ranges. (**a**) The distribution of HU and liver blood vessels within the CT scan. (**b**) Image with four HU ranges applied, (**A**) original image, (**B**) mask, (**C**–**F**) HU windowed images of [0, 400], [50, 300], [100, 200], and [150, 100], respectively.

Unlike general RGB images, CT images use a quantitation measure called the Hounsfield Unit (HU) [24]. HU is the amount of X-ray energy that is absorbed in proportion to the density of tissue when X-rays pass through the human body and has a wide range of values, from −1000 for air to 2000 for bone. In clinical practice, radiologists acquire and analyze images that can focus on the desired lesion through HU windowing that adjusts the level and width values. Motivated by this, we proposed a multi-HU windowing augmentation method that generates data with different HU ranges, including the HU range of liver blood vessels adjusted for training on images of varying brightness. Figure 2a shows the HU distribution, including the liver and blood vessels. In order to reduce the calculation influence of tissues other than the liver and blood vessels in CT images, the HU range is usually adjusted to a level of 0 and width of 400 (expressed as [0, 400]) [20]. According to the proposed augmentation method, four data ranges were used for training, which adjusted the level and width from the original CT image to [0, 400], [50, 300], [100, 200], and [150, 100], respectively. This method can maintain robustness over a wide range of HUs while solving the problem of overfitting occurring only under certain HU conditions when applying general augmentation methods. Figure 2b is an example of augmentation under different level and width conditions.

### 3.2. Encoder Combining ResNet-50 Vision Transformer

**Figure 3:**
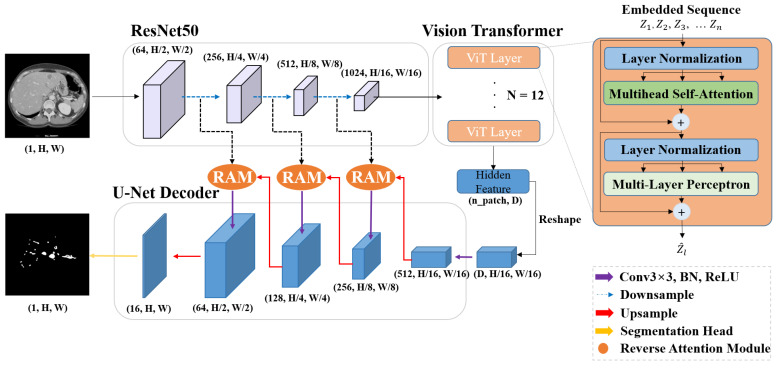
Proposed architecture. The encoder is a hybrid structure of ResNet50, a CNN feature extractor, and vision transformer. Upsampling is performed following the decoder structure of U-Net. The feature map generated by the CNN feature extractor is connected to RAM rather than skip-connected to the decoder.

The encoder structure of the proposed model is a CNN-Transformer Hybrid architecture, with ResNet50 as the CNN and Vision Transformer (ViT) as the transformer consisting of twelve encoder layers (Figure 3). First, to extract high-resolution CNN feature maps, four down-sampling layers of the ResNet50 model were used from the input layer to the last layer of each Conv Block. To align the feature map sizes during the model operation and patch splitting process, the first four Conv Block of ResNet50 were used. Specifically, the output from the 40th layer of ResNet50 is used, and, as shown in Figure 3, the output feature map size of the encoder becomes (H/16, W/16). The final extracted feature map is flattened to 1D through a linear projection (Z) process and input into the transformer’s encoder in the form of a patch. The feature map is divided into N patches (N = H × W/P^2^), which are P × P patch sizes, and each patch is position-embedded to learn location information along with context information, as shown in the following equation:

z^0^ = [x^1^_p_E; x^2^_p_E;…; x^N^_p_E] + E_pos_ (1)

where E is the patch embedding projection matrix with dimensions (P^2^·C) × D, where P^2^·C represents the size of a vectorized patch and D is the hidden size of the Transformer. E_pos_ is the positional embedding matrix that contains the location information of the patch with dimensions N × D. This alignment ensures that the patch embeddings and positional embeddings are summed elementwise for input to the Transformer encoder. The Transformer encoder consists mainly of Multihead-Self Attention (MSA) and Multi-Layer Perceptron (MLP). Embedded patches are semantically represented by applying the Self-Attention mechanism through MSA. Specifically, in MSA, the embedded patch is linearly transformed to obtain the query, key, and value, and the attention value is calculated by matrix multiplying each vector by the learning weight. Through the Multi-Layer Perceptron (MLP), which consists of two hidden layers and a GeLU function, it is possible to learn the complex patterns and associations of each patch. Transformer is stacked in 12 layers, and the output $\hat{Z}$ (n_patch, hidden size) of the last layer can represent the semantic features of fine vessels. The height and width of the original image, patch size, and hidden size used in the experiment were set to 256, 16, and 768, respectively.

### 3.3. Decoder with Reverse Attention Module (RAM)

**Figure 4:**
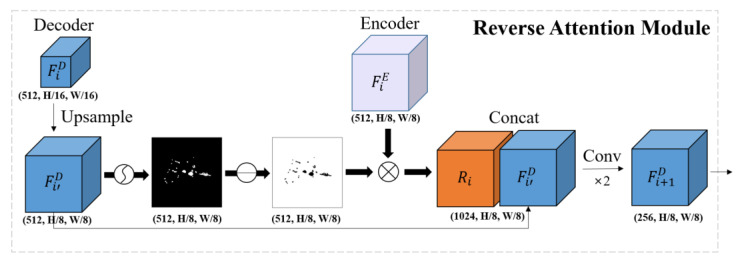
Reverse attention module. The operation of the first RAM was provided as an example. An upsampled high-level feature map $F_{i\text{’}}^{D}$ entered RAM and highlighted the edge region through reverse computation. R_i_ was generated through element-wise multiplication with a low-level feature map $F_{i}^{E}$ from Encoder. Afterwards, $F_{i\text{’}}^{D}$ and R_i_ were concatenated to output a feature map with both edge context and localization information. The concatenated feature map is then processed through two convolution operations.

Because of the global context extraction of Transformer, semantic features can be created. However, there is a limitation in that a lack of inductive bias affects the extraction of detailed spatial information. Therefore, high-resolution feature maps extracted from Encoder’s CNN were combined with upsampled features for detailed localization information. Furthermore, we introduced a Reverse Attention Module (RAM) that could focus on the edge features of liver vessels. The key difference from other methods is that RAM was applied to convey the edge features of vascular structures during the upsampling and skip connection part of the encoder-decoder architecture. As shown in Figure 4, the feature map derived from high level decoder undergoes upsampling to achieve spatial alignment with the low-level feature map F_i_ from the encoder. The upsampled feature map F_i+1_ is passed through a sigmoid activation function to generate a probability map. The probability map is inverted by applying reverse computation (1-probability value) to distinguish the background from vessel regions. The inverted feature map and the low-level feature map F_i_ from the encoding path are element-wise multiplied, enhancing spatial details by combining high-level contextual information with low-level features. The resulting refined feature map R_i_ is then concatenated with the original upsampled high-level feature map F_i+1_, producing a feature map enriched with both structural context and edge localization. The concatenated feature map is then processed through two convolution layers to integrate information and adjust the filter size. In the last layer of the proposed model, 1 × 1 conv was used to generate the final segmentation mask.

### 3.4. Evaluation Metrics

We used four performance evaluation metrics, including DSC (dice similarity coefficient), precision, sensitivity, and specificity, and evaluated them using the following Equations (2)–(5). The DSC, which calculates the intersection of the predicted value and the ground truth region, means that the closer the value is to 1, the more exactly segmented the vessel. Precision is the proportion of actual blood vessels that the model predicts are positive. Sensitivity is the proportion at which the model predicted a blood vessel among those that are actual blood vessel pixels, and a high sensitivity value means that the rate of erroneous detection of blood vessels is low. Specificity is the proportion of the model’s prediction of the background from the actual background pixels. These metrics provide a clear basis for comparison with previous state-of-the-art (SOTA) studies. To ensure comparability, we adhered to the evaluation metrics used in prior research. We defined the four evaluation metrics as:

DSC = 2∑_i_^N^ p_i_g_i_/(∑_i_^N^ p_i_^2^ + ∑_i_^N^ g_i_^2^) (2)

Precision = TP/(TP + FP)(3)

Sensitivity = TP/(TP + FN)(4)

Specificity = TN/(FP + TN)(5)

where p_i_ is the segmentation result predicted by the model, g_i_ is ground truth, and TP, FP, FN, and TN are the numbers of true positives, false positives, false negatives, and true negatives, respectively.

## 4. Experiments and Results

### 4.1. Datasets and Experiment Settings

We used 3Dircadb [36], a representative public dataset for hepatic vascular segmentation. The dataset includes contrast-enhanced CT scans of 20 patients, consisting of 10 women and 10 men; of which 75% have liver tumors. The image resolution was 512 × 512 pixels, and the number of slices per patient ranged from 74 to 260, provided in DICOM format. CT images were all of the venous phase, the pixel spacing is 0.56–0.87mm, and the slice thicknesses were 1–4 mm.

Four steps were employed for image pre-processing. First, the HU windowing enhancement was used to process data with a range of [0, 400], [50, 300], [100, 200], and [150, 100], respectively. Second, because the CT scans’ fields of view (FoV) for the 20 patients were different, they were unified through 1 mm × 1 mm resampling. Third, due to memory limitations, all images were resized to 256 × 256, and, finally, we divided 3D images into 2D slices and used a total of 17,072 slices. The vessel mask corresponding to each image slice included artery, portal vein, and venous system (or vena cava).

Due to the limited size of the dataset, the dataset was split into training and test datasets in a 4:1 ratio for a total of 20 CT scans without validation datasets. The model performance was verified through 5-fold cross-validation. Accordingly, on average, 13,658 slices were used as training datasets and 3414 slices as test datasets in each fold. The best model was selected based on the highest dice score obtained during cross-validation.

We used the Dice loss function to increase the overlapping area between predicted image and ground truth, and defined this function as:

Dice Loss = 1 − {2∑_i_^N^ p_i_g_i_/(∑_i_^N^ p_i_^2^ + ∑_i_^N^ g_i_^2^)} (6)

where p_i_ is the segmentation result predicted by the model and g_i_ is groundtruth. The larger the value of Dice loss, the greater the difference between the predicted vessels and the actual vessels, so it was trained in the direction of minimizing loss in the learning process. The proposed model trained with Adam Optimizer with a learning rate of 0.00001, batch size of 8, and epochs of 70. All experiments were conducted using NVIDIA GeForce RTX 3090 GPU, Tensorflow-gpu 2.4.0.

### 4.2. Comparison of Augmentation Methods

**Figure 5:**
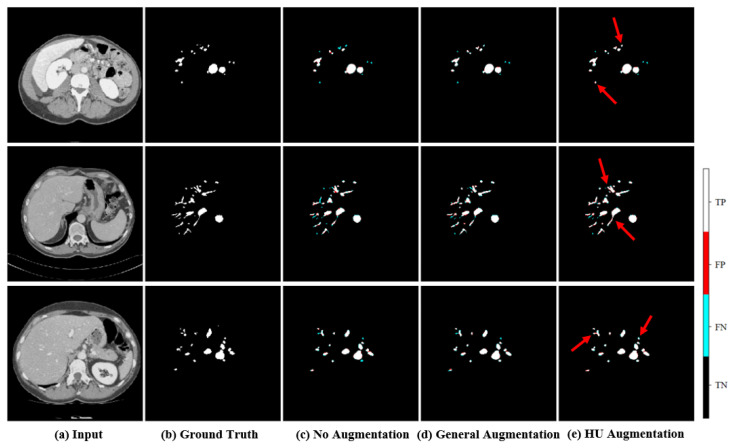
Comparison of qualitative results according to data augmentation method, (**a**) original image, (**b**) ground truth, (**c**) predicted image of model without augmentation, (**d**) predicted image of the model with augmentations such as flip, rotation, and mirroring, (**e**) predicted image of the proposed model. The red arrows indicate the regions where HU augmentation method captures TP better compared to other augmentation methods.

A lack of data to train the model can lead to overfitting problems. To compensate in this situation, data are usually augmented by transforming images using flip, rotation, and mirroring. However, the proposed method can learn under various HU ranges to maintain high segmentation performance even with images of different brightness and contrast. The segmentation performance results comparing different augmentation methods are shown in Table 1. The experimental results are based on the test dataset. The DSC of the proposed Multi-HU windowing augmentation method reached 0.948 and is 3.4% higher than the method without augmentation. In addition, it can be seen that the proposed augmentation method improved DSC by 1.2% compared to general augmentation by flip, rotation, and mirroring. We compared the qualitative results of the data augmentation method experiment and the ablation study through error maps. In the error map, black indicates non-vascular areas, cyan indicates false negatives, red indicates false positives, and white indicates vascular areas. As shown in Figure 5, the model without augmentation did not predict vessels well. HU windowing augmentation performed better on fine vessel segmentation than augmentation with flip, rotation, and mirroring.

Additionally, we compared DSCs in different HU ranges to see if the model is robust at various brightnesses, as shown in Table 2. The test dataset was used to evaluate the robustness by HU ranges. While the general augmentation method such as flip, rotation, and mirroring decreases the segmentation performance as the HU range narrows, the HU windowing augmentation method remained robust because it learned on data of various brightness. Accordingly, the proposed augmentation method was demonstrated to be an effective method for improving the performance and robustness of the model.

### 4.3. Ablation Study

**Figure 6:**
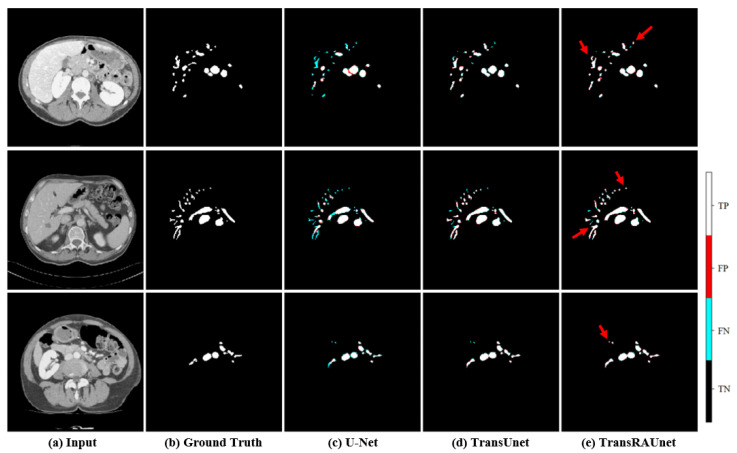
Comparison of qualitative results from the ablation study. (**a**) original, (**b**) ground truth, (**c**) UNet, (**d**) TransUNet, (**e**) the proposed model, and each predicted image is shown, respectively. The red arrows indicate the regions where TransRAUnet architecture outperforms other methods in TP detection.

**Figure 7:**
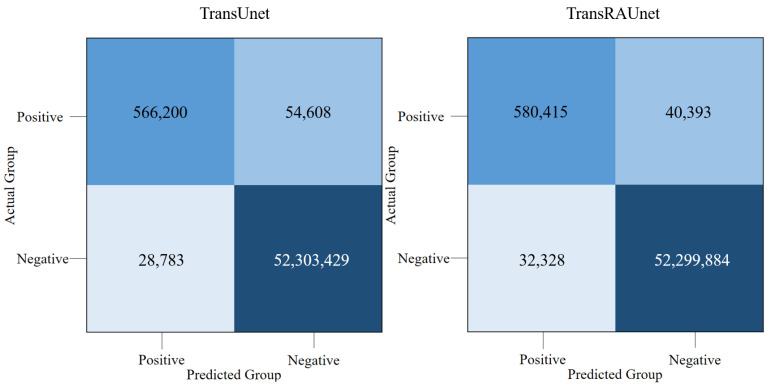
Comparison of confusion matrix results for ablation studies. (**Left**) Confusion matrix of TransUnet; (**Right**) confusion matrix of TransRAUnet.

The proposed model can reduce the false negative area because it focuses on the edge information of liver vessels. The ablation study results are based on using TransUnet with HU augmentation as the baseline. To confirm the effectiveness of RAM, we compared the quantitative results of TransUnet and TransRAUnet, as shown in Table 3. The results of the experiments were obtained using the test dataset. The DSC and sensitivity of TransRAUnet were 0.948 and 0.944, which were 0.9% and 1.7% higher than the baseline TransUnet, respectively. As shown in Figure 6, U-Net often saw fragments when segmenting large blood vessels and did not identify fine blood vessels well. Compared to TransUnet, the proposed model shows superior performance on fine vessel segmentation, and it can be seen that there were relatively few false negatives. A comparison of the confusion matrix of the two models is shown in Figure 7, and it can be seen that the false negative rate of the proposed model decreased, and the true positive rate increased. Therefore, we demonstrated that the proposed model which learned vascular edge information by introducing RAM to the baseline improved segmentation performance.

### 4.4. Comparison with State-of-the-Art Methods

To compare the performance of the proposed model, we selected state-of-the-art architectures (SegNet, UNet, UNet++, UNet3+, TransUNet, TFNEdge) and compared DSC, precision, and recall, respectively [21]. The performance of the proposed model and the other six segmentation models is shown in Table 4. The proposed model improved DSC and sensitivity by 3.5% and 6.4%, respectively, compared to UNet. When compared to the TFNEdge model that combines Transformer, CNN, and Edge Module, DSC and sensitivity improved by 2.7% and 4.3%, respectively. In conclusion, the proposed model demonstrated superior performance compared to other state-of-the-art methods.

**Table 4:** Comparison of SOTA methods in 3Dircadb dataset.

Methods	DSC	Precision	Sensitivity
SegNet [37]	0.907	0.938	0.879
Unet [17]	0.913	0.951	0.880
UNet++ [18]	0.904	0.934	0.879
UNet3+ [19]	0.917	0.945	0.894
TransUNet [23]	0.913	0.944	0.885
TFNEdge [22]	0.921	0.947	0.901
Proposed Model	0.948	0.955	0.944

## 5. Discussion

We have proposed a new architecture called TransRAUnet which improves segmentation performance on the detailed structures of liver vessels and maintains robustness under various image brightness conditions. The global context of the detailed structure of liver blood vessels was extracted with a TransUnet-based model, and edge features of blood vessels were accurately detected and segmented using the reverse attention module (RAM). In addition, we proposed a Multi-HU windowing augmentation method.

In Section 4.2, an experiment was conducted to confirm the effectiveness of the HU augmentation. When the model was compared to general augmentation methods such as rotation, flip, and mirroring, it was clearly superior. In general, data augmentation not only solves the overfitting problem by increasing the variability of the data by randomly selecting image parameters, but also improves model performance [38]. However, it has the disadvantage of poor reproducibility due to parameter randomization. On the other hand, the HU windowing augmentation method improved the performance and reproducibility of the model by variously augmenting CT images with preset HU conditions and showed that it maintained robustness even under various HU ranges. The experimental results in Table 1 and Table 2 were based on TransRAUnet, allowing us to examine the impact of HU augmentation. However, in Section 4.3, we investigated the effect of RAM. Section 4.3 describes an ablation study designed to demonstrate the results obtained in the presence or absence of RAM. The proposed model used TransUnet as a baseline, which can fuse global context extraction and local detailed information. In addition, we focused on edge features of the high-level upsampled feature map through RAM and combined it with Encoder’s low-level feature map to detect fine blood vessel structures. As shown in Figure 7, TransUnet and the proposed model captured the global context well and also showed excellent segmentation performance. In addition, the proposed model with RAM had better segmentation performance than TransUnet. Specifically, false negatives decreased, true positives improved, and the results of comparative visualization demonstrated that even fine blood vessels were detected and segmented. These results proved the effectiveness of the proposed model with the introduction of RAM.

Other state-of-the-art models designed a dense structure or used a transformer to specifically segment the context of the image. However, these models had the limitation of not being able to capture the edge feature of blood vessel walls. Meng’s TFNEdge [22] improved performance by using Canny to preserve edge information. The difference between the SOTA model and the proposed model is that we have introduced reverse attention module and trained in various HU ranges for robustness. These differences show that DSC, especially sensitivity, has improved significantly compared to the SOTA models, as shown in Table 4.

However, despite these advantages, the proposed model has two limitations. First, the number of available datasets is insufficient. 3Dircadb has only 20 CT scans, which is a small number of datasets. Furthermore, we were unable to access real clinical data due to restricted access, and there is a lack of high-quality public datasets specifically suited for hepatic vessel segmentation. To address these limitations, we evaluated generalized performance through 5-fold cross-validation to solve this problem. The second challenge pertains to the time required to generate prediction images. On average, processing a single patient’s CT scans takes around 40 s. When both quantitative results and images are produced together, the process requires approximately 1 min and 30 s. These durations are not sufficiently efficient for clinical applications. Therefore, future studies should focus on designing a model with computational speeds optimized for clinical use.

## 6. Conclusions

Prior to liver resection, clinicians must manually segment liver vessels in CT images. This is a time-consuming and difficult task due to low contrast images, noise, and the complex structure of liver vessels. Therefore, it is important to develop a method to automatically segment liver vessels within CT scans. Consequently, we have proposed TransRAUnet, a model for liver vessel segmentation that introduces RAM into TransUNet. The proposed model set TransUnet as the baseline, which is based on the U-Net structure used mainly to segment medical images. In the encoder, features were extracted by hybridizing CNN, which can connect low-level features, and Transformer, which can capably extract global context features. In Decoder, a semantic feature map was generated through RAM that can extract the edge features of the upsampled high-level feature map. Additionally, we proposed a Multi-HU windowing augmentation method to train under different HU ranges for robust performance. Specifically, the proposed model was trained by augmenting it with settings of [0, 400], [50, 300], [100, 200], and [150, 100], which are the appropriate HU ranges for liver vessels.

Through experiments, the proposed model achieved DSC 0.948 and sensitivity 0.944. The effectiveness of the proposed augmentation method was proven through comparisons with a non-augmented model and a model using a general augmentation method. Additionally, fine vascular structures were well segmented through the introduction of RAM. Lastly, it demonstrated better overall performance than current SOTA models. Furthermore, it is expected to serve as a navigation aid for liver resection surgery through accurate liver vessel and tumor segmentation.

## Figures and Tables

**Table 1:** Comparison of augmentation methods.

Methods	DSC	Precision	Sensitivity	Specificity
Augmentation (w/o)	0.914	0.950	0.887	0.999
Augmentation (w/)	0.936	0.957	0.917	0.999
Proposed Augmentation	0.948	0.955	0.944	0.999

**Table 2:** Comparison of robustness by HU ranges on augmentation methods.

Methods	[−100, 600]	[−50, 500]	[0, 400]	[50, 300]	[100, 200]	[150, 100]
Augmentation (w/o)	0.855	0.889	0.914	0.879	0.765	0.271
Augmentation (w/)	0.881	0.910	0.936	0.889	0.684	0.079
Proposed Augmentation	0.891	0.921	0.948	0.948	0.946	0.934

**Table 3:** Ablation study on the reverse attention module.

Methods	DSC	Precision	Sensitivity	Specificity
Baseline	0.939	0.957	0.927	0.999
Baseline + RA	0.948	0.955	0.944	0.999

## Data Availability

The dataset used in the experiments are publicly available at https://www.ircad.fr/research/data-sets/liver-segmentation-3d-ircadb-01/ (accessed on 14 October 2024).
